# Biodiesel biorefinery: opportunities and challenges for microbial production of fuels and chemicals from glycerol waste

**DOI:** 10.1186/1754-6834-5-48

**Published:** 2012-07-18

**Authors:** João R M Almeida, Léia C L Fávaro, Betania F Quirino

**Affiliations:** 1 Embrapa-Agroenergy, Parque Estação Biológica S/N, Av. W3 Norte (final), 70770-901, Brasília, DF, Brazil; 2 Universidade Católica de Brasília, Genomic Sciences and Biotechnology Program, 70790-160, Brasília, DF, Brazil

**Keywords:** Glycerol, Fermentation, Biofuels, Metabolic engineering, Biodiesel

## Abstract

The considerable increase in biodiesel production worldwide in the last 5 years resulted in a stoichiometric increased coproduction of crude glycerol. As an excess of crude glycerol has been produced, its value on market was reduced and it is becoming a “waste-stream” instead of a valuable “coproduct”. The development of biorefineries, *i.e.* production of chemicals and power integrated with conversion processes of biomass into biofuels, has been singled out as a way to achieve economically viable production chains, valorize residues and coproducts, and reduce industrial waste disposal. In this sense, several alternatives aimed at the use of crude glycerol to produce fuels and chemicals by microbial fermentation have been evaluated. This review summarizes different strategies employed to produce biofuels and chemicals (1,3-propanediol, 2,3-butanediol, ethanol, n-butanol, organic acids, polyols and others) by microbial fermentation of glycerol. Initially, the industrial use of each chemical is briefly presented; then we systematically summarize and discuss the different strategies to produce each chemical, including selection and genetic engineering of producers, and optimization of process conditions to improve yield and productivity. Finally, the impact of the developments obtained until now are placed in perspective and opportunities and challenges for using crude glycerol to the development of biodiesel-based biorefineries are considered. In conclusion, the microbial fermentation of glycerol represents a remarkable alternative to add value to the biodiesel production chain helping the development of biorefineries, which will allow this biofuel to be more competitive.

## Introduction

Production of biofuels and chemicals from renewable feedstocks is necessary to meet the energy demand in a world where petrol fuels are becoming scarce and more expensive. One of the main problems associated with biofuels is still the production costs, which can be reduced if residues of biofuels production processes are converted into valuable coproducts [1,2]. Biodiesel is an alternative fuel that reduces net greenhouse effects and its use has become mandatory in many countries [3]. It is mainly obtained by the transesterification of fat and vegetable oils in the presence of a catalyst by a primary alcohol (usually methanol) leading to a fatty acid methyl ester (FAME), which is used as a biofuel. Sunflower, rape, soybean and palm oils are the main substrates to make biodiesel worldwide, however, there are local variations on which is the main source. In Brazil, for example, 80% of the biodiesel produced in 2010 was from soybean oil [4].

**Figure 1:**
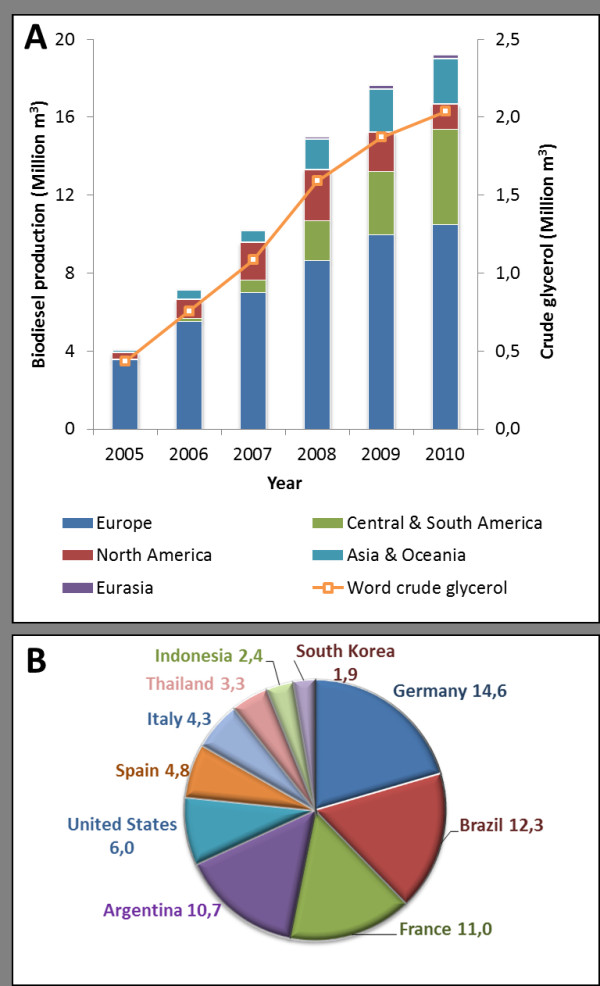
**A: World biodiesel (bars) and crude glycerol (lines) production between 2005 and 2010.** Biodiesel production was grouped by continents; whereas crude glycerol represents the total production in the world over the years. **B**: Top ten biodiesel producing countries in 2010. Their production corresponds to approximately 71.3% of the total 19.21 million cubic meters of biodiesel. Production percentage is shown for each country. The production of crude glycerol was estimated assuming 0.106 L of crude glycerol per liter of biodiesel. The above figures were derived from an interactive table generated on January 11, 2012 from U.S. Energy Information Administration, International Energy Statistics, Biofuels Production ( http://www.eia.gov/cfapps/ipdbproject/iedindex3.cfm?tid=79&pid=81&aid=1&cid=regions,&syid=2005&eyid=2010&unit=TBPD).

Biodiesel production increased considerably in the past few years and so did the amount of residues generated during its production (Figure 1A). Europe is still the biggest biodiesel producer, whereas Brazil had the highest increase in production rate in the last years when compared with United States and Europe, *i.e.* from 736 in 2005 to 2,400,000 m^3^ in 2010 (Figure 1B). Production of the two main types of residues, pies and crude glycerol, is increasing concurrently with the biodiesel industry. Pies, which are produced by pressing of palms, seeds and others for oil extraction, are usually used as feed for animals or as fertilizers, consequently adding value to the biodiesel production chain. Crude glycerol, which is derived from the transesterification reaction of fat and vegetable oils (triglycerides) to produce biodiesel, contains methanol, salts, soaps and water as the main contaminants. Concentration and presence of each contaminant will vary drastically from one industry to another, due to a variety of parameters, including oil source and reaction conditions. For instance, glycerol and water content can vary, respectively, from 92% and 6% [5] to 65% and 26% [6] in crude glycerol samples. The presence of these impurities in crude glycerol samples is expected to influence negatively the bioconversion process of this coproduct. However, it is important to note that the excess of crude glycerol produced in the biodiesel industry is leading to a decrease in glycerol prices and glycerol is now considered a waste instead of a coproduct [2]. The production of crude glycerol follows the increasing biodiesel production, since the stoichiometry of the reaction dictates that for each 10 tons of FAME, 1 ton of crude glycerol is formed (Figure 1). Thus, the development of biorefineries based on crude glycerol is expected to favor the biodiesel industry economy, by reducing costs associated with the disposal of residues and increasing production of value-added chemicals [7]. 

In this review, we discuss the strategies to produce fuels and chemicals of biotechnological interest by microbial fermentation of glycerol. We highlight naturally occurring and engineered bacteria, yeast and filamentous fungi able to produce specific chemicals, as well as the strategies to improve their performance during fermentation. Market and industrial applications of these microbial fermentation products are also discussed.

### Microbial fermentation of glycerol

**Figure 2:**
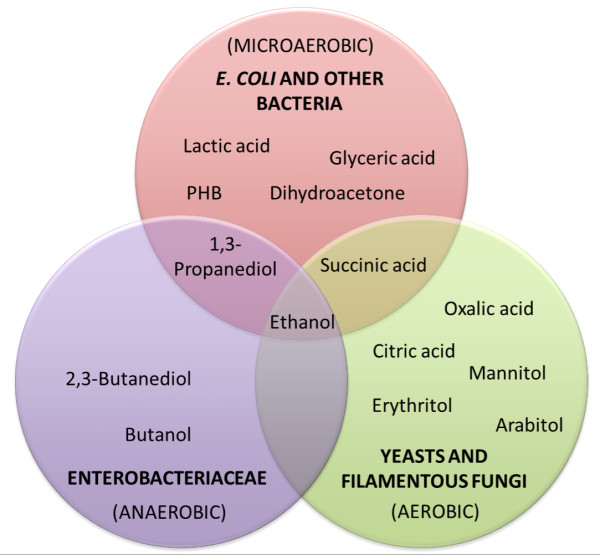
**Examples of chemicals produced by microbial fermentation of crude glycerol.** Circles/positions indicate the aerobiose conditions in which these chemicals can be produced by microbial fermentation and the main microbial producing groups.

The excess of waste glycerol produced in the biodiesel industry (Figure 1) may be used in biotechnological processes to produce value-added chemicals to avoid waste disposal and increase process economy. Due to the reduced nature of the glycerol molecule, microorganisms are able to convert it to a series of metabolites (Figure 2), with yields similar to the ones obtained when using sugars as substrates [2]. Thus, the valorization of the glycerol waste stream through the production of microbial value-added metabolites via fermentative processes has been substantially evaluated. Yeasts and filamentous fungi have been tested mainly aerobically for the production of organic acids and polyols (Figure 2). On the other hand, production of metabolites by bacteria, especially from the Enterobacteriaceae and Clostridiaceae families, such as *KlebsiellaEnterobacter, Clostridium*, has been tested under anaerobic conditions. These bacteria have been evaluated for the production of different chemicals, including the alcohols 1,3-propanodiol, 2,3-butanediol, butanol, and others (Figure 2). Engineered *E. coli* strains have been used especially under microaerobic conditions for the production of several chemicals. Biotechnological applications of these metabolites and how their production by microbial fermentation of glycerol has been optimized by metabolic engineering and fermentation strategies with different producing strains are summarized and discussed below. 

### Alcohols

#### 1,3-Propanediol

The most studied route of biotechnological valorization of glycerol is related to its conversion to 1,3-propanediol (1,3-PDO). However, commercial production of 1,3-PDO from glycerol has not been reported. 1,3-PDO can be utilized for the synthesis of the modern polymer polytrimethylene terephthalate (PTT) that in turn can be used to make carpets (Corterra®, Shell), special textile fibers (Sorona®, DuPont), monofilaments, films, and nonwoven fabrics. PTT is also used in the engineering thermoplastics area [8]. The demand of polybutylene terephthalate (PBT), another potential 1,3-PDO derivative, which is mainly consumed in the automotive and electronic sectors, in 2005 was estimated at about 376 000 tons with an average annual growth rate of 5 percent [9].

**Figure 3:**
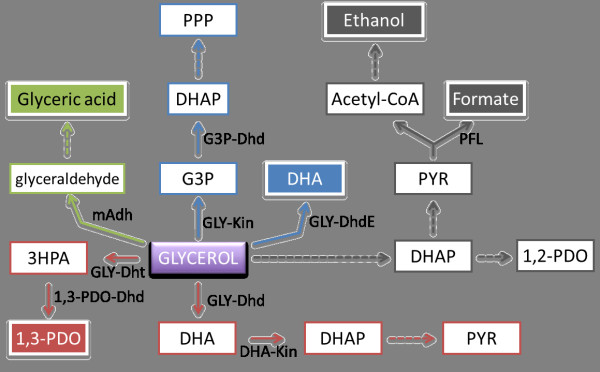
**Different metabolic pathways for metabolism of glycerol.** Production of 1,3-PDO by Enterobacteriaceae family members is shown in red. DHA production by *G. oxydans* is shown in blue. Ethanol and formate production pathways in the fermentative utilization of glycerol by *E. coli* are shown in gray. Proposed pathway for the conversion of glycerol to glyceric acid is shown in green. Dashed lines indicate multiple steps or unknown enzyme(s) (GA pathway). Main products are highlighted in color- filled boxes. Abbreviations: GLY, glycerol; GLY-Dhd, GLY dehydrogenase; DHA, dihydroxyacetone; DHA-Kin, DHA kinase; DHAP, DHA phosphate; PYR, pyruvate; GLY-Dht, GLY dehydratase; 3HPA, 3-hydroxyapropionaldehyde; 1,3-PDO-Dhd, 1,3-PDO dehydrogenase; GLY-DhdE, membrane-bound GLY-Dhd; GLY-Kin, GLY kinase, G3P, glycerol 3-phosphate, G3P-Dhd, G3P dehydrogenase; PPP, pentose phosphate pathway; PFL, pyruvate formate-lyase; mAdh: membrane-bound alcohol dehydrogenase.

PDO can be successfully produced fermentatively from glycerol by bacteria of the Enterobacteriaceae and Clostridiaceae families, mainly *Klebsiella* spp. and *Clostridium* spp., respectively [2,10,11]. Two pathways are necessary for the conversion of glycerol to 1,3-PDO under anaerobic conditions by Enterobacteriaceae (Figure 3). In the oxidative pathway, glycerol is dehydrogenated by a NAD-dependent glycerol dehydrogenase (GLY-Dhd) to dihydroxyacetone (DHA), which is then phosphorylated by phosphoenolpyruvate (PEP) and ATP-dependent DHA kinase (DHA-Kin). In the parallel reductive pathway, glycerol is dehydrated by the coenzyme B12-dependent glycerol dehydratase (GLY-Dht) to 3-hydroxypropionaldehyde (3HPA), which in turn is reduced to the major product 1,3-PDO by the NADH-dependent 1,3-PDO dehydrogenase (1,3-PDO-Dhd), thereby regenerating NAD^+^ (Figure 3)(as reviewed in [2,10,11]). 

Crude glycerol consumption and 1,3-PDO production are influenced by the purity and concentration of the glycerol, as well as by fermentation conditions [6,12,13]. For instance, higher glycerol concentrations and microaerobic conditions increases the substrate consumption and 1,3-PDO productivity, however, without affecting fermentation yield [6,13]. It was also shown that addition of fumarate at low concentrations (*i.e.*, 5 mM, equivalent to 2.9 g/L) during glycerol fermentation increased in 35% glycerol consumption and 1,3-PDO production rates without affecting product yield [14]. Several genetic engineering strategies have been employed to produce 1,3-PDO in both native and non-native microbial producers (reviewed in [10]). Regarding native producers, mainly *K. pneumoniae*, increased 1,3-PDO production has been attempted by overexpression of genes directly involved in the 1,3-PDO biosynthesis pathway and inactivation of genes involved in byproduct formation. Regarding non-native producers, a 1,3-PDO biosynthesis pathway has been constructed in bacteria and yeasts that are not naturally able to produce 1,3-PDO by heterologous overexpression of genes from natural producers [10,15,16]. Among the engineered strains, 1,3-PDO production from glycerol using *K. pneumoniae* and *E. coli* strains looks the most promising, as shown by the high 1,3-PDO titers obtained (Table 1). 

**Table 1:** Chemicals produced at high yield and/or high concentration by microbial fermentation of glycerol

**Product**	**Organism**	**Fermentation mode**	**Oxygen availability**	**Yield (product/glycerol)**	**Productivity**	**Product concentration**	**Ref.**
**1,3-Propanediol**	*K. pneumoniae* DSM 2026	Fed-batch	Microaerobic	0.52 mol/mol	1.57 g/L/h	59.50 g/L	[13]
	*K. pneumoniae* LDH 526	Fed-batch	Aerobic	0.52 mol/mol	2.13 g/L/h	102.1 g/L	[17]
	*C. butyricum* F2b	Batch	Anaerobic	0.53 g/g	1.05 g/L/h^a^	47.1 g/L	[6]
	*E. coli* K12	Fed-batch	Anaerobic	90.2%^b^	2.61 g/L/h	104.4 g/L	[16]
	*K. pneumoniae*	Fed-batch 1 m^3^	Anaerobic	61 mol/mol	2.2 g/L/h	75 g/L	[18]
**2,3-Butanediol**	*K. pneumoniae* G31	Fed-batch	Microaerobic	0.36 mol/mol	0.18 g/L/h	49.2 g/L	[19]
	*K. pneumoniae* G31	Fed-batch	Aerobic	0.39 g/g	0.47 g/L/h	70.0 g/L	[20]
**Ethanol**	*E. coli* SY 4	Batch	Microaerobic	85%^b^	0.15 g/L/h	7.8 g/L	[21]
**Butanol**	*C. pasteurianum*	Batch	Anaerobic	0.36 g/g	n.d	1.8 g/L^a^	[22]
**Dihydroxyacetone**	*G. oxydans* ZJB09112	Fed-batch	Aerobic	88.7%^b^	n.d	161.9 g/L	[23]
**Glyceric acid**	*G. frateurii* NBRC103465	Fed-batch	Aerobic	0.76 g/g	0.81 g/L/h^a^	136.5 g/L^c^	[24]
	*A. tropicalis* NBRC16470	Fed-batch	Aerobic	0.46 g/g	0.71 g/L/h^a^	101.8 g/L^d^	[24]
**Lactic acid**	*E. coli* AC-521	Fed-batch	Aerobic	0.9 mol/mol	0.49 g/g/h^a^	85.8 g/L	[25]
	*E. coli* LA02Δdld	Batch	Microaerobic	0.83 g/g	1.25 g/g//h	32 g/L	[26]
**Succinic acid**	*engineered E. coli*	Batch	Microaerobic	0.69 g/g	~ 4 g/g/h	14 g/L	[27]
	*Y. lipolytica* Y-3314	Batch	Oxygen limited	0.45 g/g ^d^	n.d	45 g/L	[28]
**Citric acid**	*Y. lipolytica*	Repeated batch	Aerobic	0.77 g/g	0.85 g/L/h	124.2 g/L	[29]
**Oxalic acid**	*A. niger*	Batch	Aerobic	0.62 g/g	n.d	21 g/L	[30]
**Mannitol**	*C. magnoliae*	Batch	Aerobic	0.51 g/g	0.53 g/L/h^a^	51 g/L	[31]
**Erythritol**	*Y. lipolytica* Wratislavia K1	Fed-batch	Aerobic	0.56 g/g	1.0 g/L/h	170 g/L	[32]
**Arabitol**	*D. hansenii* SBP1	Batch	Aerobic	0.50 g/g	0.12 g/L/h^a^	14 g/L^a^	[33]
**PHB**	*E. coli Arc2*	Fed-batch	Microaerobic		0.18 g/L/h	10.81 g/L	[34]
	*Z. denitrificans* MW1	Fed-batch	Aerobic	0.25 g/g	1.09 g/L/h	54.3 g/L	[35]

^a^ calculated from the data presented; ^b^ Percentage of theorethycal maximum; ^c^ 72% D-GA enantiomeric excess (ee); ^d^ 99% D-GA enantiomeric excess (ee); n.d. not determined.

#### 2,3-Butanediol

2,3-Butanediol (BDO) can be employed in many chemical syntheses. For instance, it can be used to produce plastics, anti-freeze solutions and solvent preparations. In addition, it can be converted to methyl ethyl ketone (a liquid fuel additive), 1,3-butadiene (used to produce synthetic rubber), diacetyl (a flavoring agent), or to precursors of polyurethane (used in the pharmaceutical and cosmetics industries) [36,37]. BDO is chemically obtained from petroleum; however, the development of a microbial production route based on renewable feedstocks is of interest. Fermentation of glycerol by several strains of *Klebsiella* spp. [11,12,18], resulted in BDO production. However, the main product of such fermentations is 1,3-PDO, whereas BDO is a minor product along with acetate, lactate, succinate, and ethanol.

Recently, a study demonstrated that BDO can be obtained as a major product of glycerol fermentation by *K. pneumoniae* G31 [19]. Strain specificity and culture pH were the main factors influencing BDO production. Indeed, alternate production of BDO and acetic acid, the second most produced metabolite by *K. pneumoniae* G31 were demonstrated to be associated with pH self-control; i. e. during fermentation the microorganism alternates production of BDO and acetic acid to adjust pH of the cultures. In addition, BDO production was influenced by medium composition and aeration. When medium composition, aeration regime, and the initial pH of the fermentation were optimized, the production of BDO reached 70 g/L, the highest concentration obtained from glycerol, with a maximum yield of 0.39 g/g glycerol (Table 1) [20].

### Ethanol

Ethanol is mainly used as fuel for transportation, but it can also be utilized in industry as a solvent and chemical intermediate. World fuel ethanol production, mainly based on yeast fermentation of sugarcane sucrose and corn starch, has reached 114 millions of cubic meters in 2011 [38]. In addition, the development of technologies for ethanol production based on lignocellulosic feedstocks has been evaluated intensively [39,40] and should also contribute to the increasing demand for this fuel. On the other hand, ethanol production from glycerol has gained modest attention in the last years.

Bacteria of the Enterobacteriaceae family and of the *Clostridium* genus are able to convert glycerol to ethanol, however, yields are relatively low because ethanol is only a secondary product of the fermentation [18,19,41]. In these cases, the main product of fermentation is 1,3-PDO [18,41] or 2,3-butanediol [19]. For instance, during pilot scale production of 1,3-PDO, concentrations of ethanol from glycerol varied from 8 to 17 g/L during fed-batch fermentation of *K. pneumoniae*[18,41].

Recently it was demonstrated that *E. coli* can convert glycerol to ethanol anaerobically [42], as well as aerobically [21] (Figure 3). Initially, it was demonstrated that *E. coli* is able to ferment glycerol anaerobically in a pH-dependent manner, which is linked to CO_2_ availability. Glycerol fermentation proceeds under acidic conditions (pH6), however it is impaired under alkaline conditions (pH8) because CO_2_ availability is reduced (*i.e.*, most CO_2_ is converted to bicarbonate) [42]. Afterwards, several fold improvements in the production of ethanol and also of the coproducts hydrogen and formate was achieved by genetic engineering [43]. Byproduct formation was reduced in the strains producing ethanol-hydrogen and ethanol-formate by mutations that inactivated fumarate reductase (*DfrdA*) and phosphate acetyltransferase (*Dpta*). To prevent the conversion of formate to CO_2_ and H_2_, the strain producing ethanol–formate also contained a mutation that inactivated formate–hydrogen lyase (*DfdhF*). Finally, high rates of glycerol utilization and product synthesis were obtained by simultaneous overexpression of glycerol dehydrogenase (*gldA*) and dihydroxyacetone kinase (*dhaKLM*). The production of ethanol from glycerol attained a yield of 85% of the theoretical maximum (the maximum theoretical yield is 1 mol each of H_2_ and ethanol per mol of glycerol fermented) in both strains [43]. Fermentation performances of these strains were further improved by elimination of lactate production by deletion of *ldhA*, which encodes a lactate dehydrogenase, followed by fermentation under microaerobic conditions [21]. Conversion rates of glycerol to ethanol as high as 85% of the theoretical maximum in mineral medium were obtained (Table 1). These results highlight the potential for using *E. coli* as a host for the production of ethanol from glycerol.

Production of ethanol from glycerol by the methylotrophic yeast *Hansenula polymorpha* was recently evaluated [44]. A recombinant *H. polymorpha* yeast strain expressing the *pdc* and *adhB* genes, which encode, respectively, pyruvate decarboxylase and aldehyde dehydrogenase II, from *Zymomonas mobilis* exhibited a 3.3-fold increase in ethanol yield from glycerol fermentation under microaerobic conditions. However, the final concentration of ethanol obtained was still very low, *i.e.* 2.74 g/L [44].

### Butanol

Butanol has been identified as an alternative fuel and as a key chemical platform that can be industrially converted to acrylates, ethers, and butyl acetate, which in turn are utilized in paints, lacquers, and resin formulations [45]. Butanol has been produced by *Clostridium* spp. fermentation of sugar for years. *Clostridium pasteurianum* can produce 1,3-PDO and butanol through anaerobic fermentation of glycerol [46]. However, only recently the production of butanol from crude glycerol generated during biodiesel production was evaluated [22]. *C. pasteurianum* was shown to produce butanol when grown in crude glycerol, although butanol yields and productivity on this substrate was considerably lower than on glycerol. Indeed, yields were approximately 20% higher on glycerol than in crude glycerol (from 0.36 g/g to 0.30 g/g (g butanol/glycerol consumed), whereas fermentation time was 2.5 times longer (*i.e.*, 25 days instead of 10 days) on crude glycerol. The reduced yield and productivity on crude glycerol may be caused by contaminants in the crude glycerol, which contained approximately 90–95% glycerol, 5–10% methanol and/or water, and 3–5% sodium sulfate [22]. These results indicate that *C. pasteurianum* may be used to produce butanol from biodiesel-derived crude glycerol to produce butanol, but its immediate industrial applicability is limited because of its slow growth and low butanol productivity.

### Ketone and organic acids

#### Dihydroxyacetone phosphate

Another important chemical produced from glycerol fermentation is dihydroxyacetone (DHA), the main active ingredient in all sunless tanning skincare products [47,48]. DHA also serves as a versatile building block for the organic synthesis of a variety of fine chemicals. It is produced through oxidative fermentation by *Gluconobacter oxydans* via a membrane-bound glycerol dehydrogenase (GLY-DhdE) (Figure 3) [47,48]. This appears to be the only reaction responsible for DHA synthesis and employs oxygen as the final acceptor of reduced equivalents, without NADH involvement. While DHA is produced by a GLY-DhdE, growth of *G. oxydans* on glycerol is ensured by a cytoplasmic pathway. In this pathway glycerol is phosphorylated to glycerol-3-phosphate (G3P) and then dehydrogenated to DHAP by glycerol kinase (GLY-Kin) and G3P-Dehydrogenase (G3P-Dhd), respectively. Finally, DHAP is catabolized in the pentose phosphate pathway (PPP) (Figure 3).

*G. oxydans* fermentation of glycerol for the production of dihydroxyacetone (DHA) is a process used in industry, however, there are problems related to microbial DHA production [49]. For example, increased concentrations of glycerol inhibit DHA production and bacterial growth. In addition, production of glyceric acid (GA) by membrane-bound alcohol dehydrogenases (adhA) reduces production of DHA. To overcome these problems, a strain with disrupted *AdhA* with improved ability to grow in a higher concentration of glycerol (220 g/L) and to produce DHA compared to a wild-type strain (*G. oxydans* NBRC 12528) was generated. While the wild-type strain produced 38 g/L of DHA and 47 g/L GA when cultivated in 150 g/L of glycerol, the *AdhA*-disrupted strain produced only 2.2 g/L of GA and increased production of DHA to 108 g/L [49]. Recently, an independent study demonstrated that optimization of growth medium and fermentation conditions to increase glycerol conversion to DHA by *G. oxydans* ZJB09112 lead to the production 161.9 g/L of DHA at a conversion rate of 88.7% in a fed-batch process (Table 1) [23].

### Glyceric acid

While glyceric acid (GA) coproduction in the DHA production process is a problem, GA production by itself is of commercial interest. GA may be used in the chemical and pharmaceutical industries as a building block and for the production of polymers and surfactants [50]. However, GA is not bulk produced, probably because a sizable number of applications for this chemical have not yet been developed. Because the GA molecule has three functional groups, it has a huge potential as a chemical that will add value to glycerol.

Like DHA, GA is mainly biotechnologically produced by bacteria, more specifically by the family of acetic acid bacteria (Acetobacteraceae), such as *Gluconobacter* sp., *Acetobacter* sp., and *Gluconacetobacter* sp. A membrane-bound Adh was shown to be involved in GA production by acetic acid bacteria (Figure 3) [24]. Recently, the ability of 162 acetic acid bacterial strains to produce glyceric acid was evaluated regarding productivity and enantiomeric composition of the product [24]. Productivity of glyceric acid varied from less than 10 g/L up to 40 g/L, whereas enantiomeric purity varied from less than 70% up to 99%. After optimization of glycerol concentration and aeration, two selected strains, *Gluconobacter frateurii* NBRC103465 and *Acetobacter tropicalis* NBRC16470, were able to produce more than 100 g/L of glyceric acid in fed-batch fermentation. *G. frateurii* NBRC103465 produced 136.5 g/L of glyceric acid with a 72% D-GA enantiomeric excess (ee), with yields of GA and DHA of 0.58 mol/mol and 0.12 mol/mol glycerol, respectively. On the other hand, *A. tropicalis* NBRC16470 produced 101.8 g/L of glyceric acid with 99% of enantiomeric excess (Table 1) [24]. Identification of pathways responsible for the conversion of glycerol in these microorganisms is expected to allow the use of metabolic engineering strategies to reduce byproduct formation and lead to an industrial producer strain [24,51].

### Lactic acid

Lactic acid has been used in the food industry for several years but has many other applications. Lactic acid can be processed to make acrylic acid or 1,2 propanediol used in polyester resins and polyurethane used as deicer or antifreeze [52]. Lactate esters are used as green solvents for coating and in the cleaning industry, it can also be polymerized into the biobased polymer PLA (poly-lactic acid). As lactic acid became an important building block in the chemical industry, its microbial production has advanced significantly. Indeed, the availability of cheap and abundant residues from the biofuels industry is leading to a shift from chemical to microbial production of lactic acid.

Lactic acid bacteria fermentation processes in the food industry for production of lactic acid has a long history. Current processes for D-lactate production based on native lactic acid bacteria use sugars as carbon source and may also be applicable to lignocellulosic feedstocks [53,54]. However, the utilization of lactic acid bacteria in industrial processes requires complex nutrients for cell growth and may not result in high product selectivity and enantiomeric purity [55].

Alternatively, lactic acid can also be produced from glycerol by other naturally producing microorganisms, including *E. coliKlebsiellaClostridiaBacillus* and the filamentous fungi *Rhizopus oryzae*[46,56-59], although at very low concentrations and productivity. To overcome these issues, new strains have been screened and natural producers have been metabolically engineered to increase yield and production rate. In a screening of soil bacteria, the strain *E. coli* AC-521 was isolated based on its ability to use glycerol as carbon source and to grow quickly under aerobic conditions. This strain was able to produce lactic acid with a productivity of 0.49 g/g/h and a yield of 0.9 mol/mol glycerol (Table 1) [25].

Only recently *E. coli* has been engineered for homofermentative production of D-lactate from glycerol [26]. Several enzymes leading to lactic acid production were overexpressed, while pathways leading to production of ethanol, succinate and acetate were blocked. The engineered *E. coli* strain was able to produce optically pure (99.9%) D-lactic acid from glycerol in minimal salts medium with only few supplements. In addition, this strain produced 32 g/L of D-lactate from 40 g/L of glycerol at a yield of 0.83 g/g glycerol, and with specific productivity for D-lactate production of 1.25 g/g cell mass/h [26].

### Succinic acid

Succinic acid is largely used for manufacturing health-related products, including pharmaceuticals, antibiotics, amino acids, and vitamins. In addition, it is an important building-block chemical that could be used to produce important precursors for chemical synthesis such as tetrahydrofuran, g-butyrolactone, 1,4-diaminobutane, 1,4-butanediol that are converted into a wide variety of products, including green solvents, pharmaceutical products, and biodegradable plastics [54]. Natural succinate-producing rumen bacteria, such as *Anaerobiospirillum succiniciproducens,* can produce succinate from glycerol [60]. But these bacterial strains require complex nutrients that increase production costs, purification, waste treatment and, consequently, hinder their utilization in an industrial process. Alternatively, the fermentative production of succinic acid from glycerol has been evaluated using either metabolically engineered *Escherichia coli*[27,61,62] or yeast strains (Table 1) [28,63,64].

Different strategies have been utilized to construct metabolically engineered *E. coli* strains for production of succinic acid from glycerol [27,61,62]. In a recent study, a strain was constructed in two steps and the use of a heterologous enzyme was needed [27]. Initially, byproducts formation was avoided by blocking pathways leading to ethanol, acetic and lactic acid. Then, *Lactococcus lactis* pyruvate carboxylase (*pyc*) was expressed in this strain to drive the generation of succinate from the pyruvate node. The resulting strain was able to convert glycerol to succinic acid with a specific productivity of ~ 4 g/g/h and a yield of 0.69 g succinate/g glycerol, which is similar to yields obtained with glucose (0.78 g/g). However, fermentation under microaerobic conditions was necessary to overcome the need for rich nutrients and keep a net production of ATP. On the other hand, the use of microaerobic conditions may be a problem because it will lead to glycerol loss in form of CO_2_[27].

In a concurrent study, *E. coli* was engineered to produce succinate from glycerol using only native genes [61]. Production of succinate was increased in 3 steps: i) coupling energy generation, *i.e.* ATP formation, to succinate production by increasing the activity of gluconeogenic phosphoenolpyruvate carboxykinase to perform the carboxylation of phosphoenolpyruvate; ii) avoiding formate and ethanol accumulation by inactivating pyruvate formate-lyase (*pflB*) and; iii) deleting *ptsI* (part of the intracellular phosphorelay system) which disrupts the primary pathway for anaerobic glycerol metabolism. With these modifications assembled, a strain able to produce succinate from glycerol with yields as high as 0.8 mol/mol glycerol was obtained. However, succinic acid productivity at anaerobic conditions was very low (*i.e.*, 102 mM or 40 g/L of succinate was produced in 6 days).

Although the engineering strategies attempted so far resulted in strains with slow metabolism and growth on glycerol and/or need of oxygen supply during fermentation, they are important because they pave the way for the development of biotechnology applications using glycerol-derived succinate.

Alternatively, succinic acid can be produced by yeast. In a recent study a recombinant strain of the aerobic yeast, *Yarrowia lipolytica,* was able to produce succinic acid when cultivated on glycerol at low pH [28]. The strain was constructed by deletion of the succinate dehydrogenase (SDH) subunit, which induces accumulation of succinic acid in *Y. lipolytica*, and also in fermentative yeasts as *S. cerevisiae* and *Kluyveromyces lactis*[28,63,64]. Unexpectedly, mutations in the succinate dehydrogenase subunit were shown to prevent *Y. lipolytica* growth on glucose, however, growth on glycerol and production of succinate was possible. Indeed, a strain with the *SDH2* gene deleted was able to produce succinate from glycerol at the level of more than 45 g/L in shaking flasks with buffering and more than 17 g/L without buffering [28].

### Citric acid

Citric acid is a weak organic acid that is commercially produced by fermentation of molasses (sucrose and glucose) by the fungus *A. niger.* As the citric acid global production has reached 1.6 million tons and keeps increasing annually at 3.5 - 4.0% in demand [65], its production from glycerol is also of interest. Among different potential producers of citric acid, the yeast *Y. lipolytica* has gained much attention in the last years as it is able to metabolize several important industrial and agro-industrial byproducts (*i.e.* saturated free fatty acids, raw glycerol) to produce large amounts of Single Cell Oil (SCO), organic acids and biosurfactant carbohydrate moieties (for instance, mannose and, rhamnose) [66]. As expected, *Y. lipolytica* is able to synthesize citric acid using different carbon sources and secret it into the medium in conditions of excess carbon and nitrogen deficiency [67].

Several groups reported citric acid production from glycerol by *Y. lipolytica* but at low production levels and yields [6,67,68]. High production levels (*i.e.*, above 100 g/L) were only obtained when better strains were selected [69,70] and the cultivation mode was improved [5,29,71]. The importance of strain selection was highlighted when 27 strains of *Y. lipolytica* were compared based on the ability to produce citric acid from glycerol. Citric acid production in a nitrogen-limited medium varied from 1.4 g/l up to 21.6 g/L according to the strain utilized [69]. Thus, identification of strains that produce not only lower levels of byproducts, such as isocitric acid and biomass, but also exhibit high yields and productivity will benefit the citric acid production process [69,70].

During batch fermentation of glycerol to citric acid, the yeast is mainly affected by high concentrations of crude glycerol, its impurities, and the increasing concentrations of citric acid present at the end of fermentation. Indeed, productivity of citric acid was shown to decrease over time during crude glycerol fermentation by *Y. lipolytica*[5,29]. To overcome these problems, at least partially, a repeated-batch strategy for the production of citric acid by *Yarrowia* using crude glycerol has been evaluated [5,29]. The crude glycerol was composed by glycerol 76% (w/w), sodium salts 4% (w/w), methanol 0.1% w/w, metals Cu 0.3, Mg 100, Fe 13.7, Zn 2.9, and Ca 46 (ppm), other organic materials 0.8% (w/w) and water 19.5% (w/w). In this setup, cultivation was conducted in batch mode, then a portion of the culture liquid was withdrawn, and the same volume of the production medium was added. With that, cells were able to keep the production of citric acid as high as 124.2 g/L with a yield of 0.77 g/g and a productivity of 0.85 g/L/h for approximately 1000 h (Table 1) [29].

### Oxalic acid

Oxalic acid is an organic acid known for its ability to leach iron oxides. It can be applied in industries, such in the manufacture of paper and detergents, to clean or bleach iron complexes [72]. Production of oxalic acid by *Aspergillus niger* growing in crude glycerol waste from biodiesel production plants was recently demonstrated [30,72]. Oxalic acid production reached approximately 21 g/L with a conversion yield of glycerol to oxalic acid (g/g) ranging from 0.55 to 0.62. These results look promising even considering the long production time of approximately 10 days. More recently, the ability of *A. niger* XP strain to produce oxalic acid was studied in submerged cultures containing 50 g/L of unpurified biodiesel-derived waste, which was composed of 45% (w/w) glycerol, 49% (w/w) free fatty acids, a low amount of fatty acid ethyl esters, and soaps from ethyl ester production [72]. After 7 days of biosynthesis, the quantity of oxalic acid produced by *A. niger* XP reached 48.9 g/L with yields of oxalic acid as high as 0.88 (g. g − 1 glycerol consumed).

### Polyols

Polyols (also called sugar alcohols, polyhydric alcohols, or polyalcohols) are carbohydrates with a carbonyl group (aldehyde or ketone) reduced to a corresponding hydroxyl group. Examples of commercially available sugar alcohols include xylitol, sorbitol, mannitol, erythritol, lactitol, maltitol, and hydrogenated starch hydrolysate (HSH) [73]. These compounds are used in a variety of applications, especially in the food, pharmaceutical, and medical industries, but they also serve as intermediates in chemical synthesis [74]. Sugar alcohols share many attributes with sugars and have unique nutritional properties. They are used to improve the nutritional profile of food products due to their low caloric content, low insulin-mediated response, and non-cariogenicity [75-79]. These compounds and their derivatives also have other industrial applications, including the production of polyurethanes, plastifying agents, resins, surfactants, and intermediates for producing hydrocarbons [80-83].

In fact, sugar alcohols – especially sorbitol, xylitol, and arabitol – have been identified as belonging to a group of the 12 best chemical building blocks derived from biomass in studies by the U.S. Department of Energy [84]. Sorbitol can be converted into chemicals such as propylene glycol, ethylene glycol, isosorbide, and anhydro sugars, whereas xylitol and arabitol can be converted into xylaric/xylonic acid, arabinoic/arabonic acid, and glycols [81,84-86]. Due to their wide variety of applications, including their use in biorefineries, the production of polyols has increased worldwide. According to a recent study [73], the global market for polyols is expected to reach 1.81 million tons by the year 2015.

Traditionally, industrial production of sugar alcohols has mainly been achieved using chemical means (Table 2), more specifically by sugar hydrogenation (*i.e.*, glucose, xylose, fructose) with chemical catalysts under high temperature and pressure [87], a process that requires pure substrates and costly chromatographic purification steps. In recent years, the need to develop new polyol production methods has arisen, and much attention has been paid to biochemical processes. In fact, polyol production by biochemical means offers the potential to produce an environmentally friendly product with high specificity and absence of impurities [74], an efficient and cost-effective approach compared to their production by chemical means. To produce sugar alcohols, fermentation processes employing glucose, fructose, and maltose as carbon sugars sources have been optimized and metabolic engineering strategies have been applied to different microorganisms [74-80,88-91]. 

**Table 2:** Market and current production processes of selected chemicals

**Product**	**Annual Production (t)**	**Production process**	**Ref.**
**1,3-Propanediol**	130,000	Petrochemical	[9]
**2,3-Butanediol**	1,250,000	Petrochemical	[92]
**Ethanol**	61,000,000	Microbial fermentation	[93]
**n-Butanol**	2,800,000	Petrochemical	[9]
**Lactic acid**	350,000	Microbial fermentation	[94]
**Succinic acid**	16 -30,000	Petrochemical	[95]
**Citric acid**	1,600,000*	Microbial fermentation	[54]
**Oxalic acid**	124,000*	Petrochemical	[54]
**Mannitol**	13,600 - 50,000	Chemical conversion of sugars	[77,78]
**Erythritol**	20,000 – 23,000	Microbial fermentation	[79,96]

*Estimated values.

Due to the high cost of using certain pure sugars (for example, xylose, fructose, erythrose) for catalytic hydrogenation in comparison to the final product (sugar alcohols), many studies have instead described the use of cellulosic and hemicellulosic biomass hydrolysates as sources of carbon for fermentative polyol production [97]. Although most sugar alcohols are industrially produced by chemical means, commercial production using microorganisms in fermentative processes is becoming a reality, as in the case for mannitol [98] and xylitol [97]. With the advent of an abundant and cheap carbon source – such as crude glycerol from biodiesel production – new efforts have sought to either microbially produce sugar alcohols from this carbon source or employ it as a complementary source to the sugars traditionally used in the industry [31,99]. Next the results from using glycerol or crude glycerol as sources of carbon for production of the polyols mannitol, erythritol, and arabitol with the aid of different microorganisms are discussed.

### Mannitol

Some factors that influence the ability of two yeasts (*Torulopsis mannitofaciens* CBS 5981 and *T. versatilis* CBS 1752) to produce mannitol from glycerol have been investigated [100]. In optimal conditions, *T. mannitofaciens* produced mannitol from glycerol consumed with a yield of 31% of the theorethycal maximum. In addition, it was shown that high concentrations of nitrogen sources and KH_2_PO_4_ in the culture medium significantly reduced mannitol yield despite consumption of glycerol. Previous studies reported *Candida magnoliae* as excellent mannitol producer using glucose and fructose mixtures as carbon sources [98]. A more recent study investigated mannitol production from glycerol using resting cells of this species [31]. It was shown that *C. magnoliae* was able to consume 100 g/L glycerol in 96 h, resulting in 51 g/L mannitol, which corresponds to a yield of 0.51 g/g. In addition, mannitol was the only metabolite detectable by UV–VIS or RI detector when samples from resting cells of *C. magnolia* were analyzed using ion exclusion high performance liquid chromatography. The absence of other metabolites in solution may facilitate mannitol recovery. Khan *et al.* (2009) also found that the mannitol yield was negatively affected when glycerol was supplemented with KH_2_PO_4_ and yeast extract.

André and co-workers [101] investigated the ability of different strains of *Yarrowia lipolytica* to convert residual crude glycerol (glycerol 70% w/w, impurities composed of potassium and sodium salts (12% w/w), non-glycerol organic material (1% w/w), methanol (2% w/w) and water (14% w/w)) from biodiesel production into chemical compounds with higher aggregate value. In this study, the strains *Y. lipolytica* LFMB 19, *Y. lipolytica* LFMB 20, and *Y. lipolytica* ACA-YC 5033 were cultured in nitrogen-limited culture medium with a residual crude glycerol concentration of 30 g/L. Under these conditions, the main metabolic product synthesized by the strains LFMB 19 and LFMB 20 was mannitol (6.0 g/L maximum quantity and yield of 0.2 to 0.26 g per g of crude glycerol consumed). This metabolite was produced in negligible quantity by the ACA-YC 5033 strain [101]. In an independent study [102], other 15 strains of different species of yeast and filamentous fungi of the class Zygomycetes were compared on their ability to convert crude glycerol into different chemical compounds. Increasing concentrations of crude glycerol (30, 60, and 90 g/L) were used to screen the strains under nitrogen-limited conditions. *Y. lipolyticaPichia membranifaciens*, and *Thamnidium elegans* were able to grow in culture medium containing high initial glycerol concentrations without inhibition by the substrate. In the case of *Y. lipolytica*, the best producer of mannitol under the conditions analyzed, there was a positive correlation between the increase in crude glycerol concentration in the initial culture medium and mannitol production, with mannitol concentration and with yields reaching 19.4 g/L and 0.23 g/g, respectively [102].

These examples demonstrate the importance of evaluating different microbial strains for mannitol production from crude glycerol to select the most promising strains. Although the production of mannitol on commercial scale by bacteria using conventional carbon sources has been accomplished [103], reports on the bioconversion of crude glycerol into mannitol by bacteria are scarce. Furthermore, the previous examples reveal the need for further studies on the biochemical events that lead to biosynthesis of mannitol and other polyols; for example, *Y. lipolytica* strains grown in residual crude glycerol.

### Erythritol

Commercial erythritol production occurs exclusively via fermentation in substrates containing sugars, such as glucose and fructose, from the hydrolysis of biomass. Although fermentative erythritol production by different microorganisms has been studied since 1960 [79,104], few studies have investigated the production of this polyol from different carbon sources [31]. In the specific case of using residual crude glycerol, containing 550 g/L of glycerol and 50 g/L of KCl, as a carbon source, an acetate-negative mutant of *Y. lipolytica* (Wratislavia K1) was found to simultaneously produce significant quantities of erythritol and citric acid [105]. With an initial crude glycerol concentration of 150 g/L in nitrogen-limited culture medium and a pH of 5.5 favorable for producing citric acid, a concentration of 81 g/L erythritol was obtained after fed-batch fermentation for 97 h.

Subsequently, the effect of pH on erythritol production by the Wratislavia K1 mutant of *Y. lipolytica* was investigated [32]. Erythritol concentration reached 170 g/L (0.56 g/g yield) after fed-batch growth with a total concentration of 300 g/L crude glycerol and pH of 3.0. Under these conditions, citric acid was not produced, demonstrating that this pH range is optimal for the mutant to produce erythritol, as it prevents the channeling of glycerol to citric acid production [32]. This example demonstrated that by using residual crude glycerol as the carbon source, the yields of erythritol without the generation of undesirable byproducts were comparable to the reported yields with microorganisms used in commercial erythritol production with glucose as substrate [32]. As previously reported for mannitol production, it is noteworthy that approaches involving bioprospecting microorganisms able to tolerate high osmolarity [106] can lead to a higher yield relative to erythritol-producing industrial strains.

### Arabitol

Arabitol polyol has many attributes of its enantiomer, xylitol, making its use feasible in many known applications of xylitol, such as in natural sweeteners, caries reducers, and sugar substitutes for diabetic patients [33]. Arabitol and xylitol can be transformed into arabinoic/arabonic and xylaric/xylonic acids, which in turn can be used to produce unsaturated polyesters and polymers with new applications [84,86]. This role indicates their importance in the biorefinery context. In addition, arabitol can be biologically converted into xylitol, for example, by *Gluconobacter oxidans*[107,108], representing a possible efficient route for the synthesis of xylitol of a higher purity and specificity.

In this scenario, Koganti [33] conducted an extensive screening of over 214 yeast strains belonging to 25 different genera from the Agricultural Research Service Culture Collection (NRRL) with regard to their ability to produce arabitol/xylitol using crude glycerol from biodiesel production. In this study, xylitol yield was low for most strains; however, the *Debaryomyces hansenii* SBP-1 strain was selected for its ability to produce high arabitol concentrations as the only polyol, thus facilitating further separation of the product. Arabitol production by *D. hansenii* SBP-1 was then investigated under different conditions, and a yield of 0.5 g/g glycerol consumed was attained with 150 g/L crude glycerol in the initial culture medium under aerobic conditions at 30°C.

Subsequently, culture conditions for the *D. hansenii* SBP-1 strain were optimized [109], leading to an arabitol yield of 0.6 g/g and volumetric productivity of 0.35 g/L/h. A protocol was developed for separating arabitol from the fermentation medium containing 20–30 g/L glycerol and 40–50 g/L arabitol, which led to a 60% arabitol yield with 95% purity.

### Others

#### PHB

Polyhydroxyalkanoates (PHAs) have received great attention due to their potential application as renewable, biodegradable, and biocompatible thermoplastics [110]. They can be completely degraded by microorganisms present in most environments, and can be produced from different renewable carbon sources [111]. Poly-3-hydroxybutyrate PHB belongs to the group of polyhydroxyalkanoates (PHAs) and it is the best-studied example of biodegradable polyesters. This polymer is naturally synthesized by a wide variety of bacterial species as a storage compound for carbon and energy [112]. Nowadays, commercial production of PHB is small and limited by substrate cost [111]. Thus, efficient conversion of cheap crude glycerol from the biodiesel industry to PHB is an interesting opportunity to reduce production costs and make PHB an industrial biotechnological product [34,35]. Indeed, microbial PHB synthesis from glycerol has been evaluated with several PHA producers and under different fermentation conditions [34,35,113-116].

Conversion of glycerol to PHBs has reached high production levels due to optimization of strains and fermentations conditions. Implementation of fed-batch under low O_2_ conditions appeared to be a suitable strategy for the production of PHB by the genetic engineered *E. coli Arc2* strain [34]. In microaerobic fed-batch cultures in which glycerol was added to maintain its concentration above 5 g/L, cell dry weight and PHB concentration reached 21.17 and 10.81 g/L, respectively, after 60 h (Table 1) [34]. Similarly, fed-batch cultivation improved PHB production by the newly isolated bacterium, *Zobellella denitrificans* MW1, which was characterized as producing large amounts of PHB from glycerol in presence of NaCl [35,114]. Cultivation in the medium containing 20 g/L NaCl, with optimized feeding of glycerol and ammonia, resulted in a PHB content of 66.9% of cell dry weight, and the polymer productivity and substrate yield coefficient of 1.09 g/L/h and 0.25 g PHB/g glycerol, respectively [35].

### D-Xylulose

Conversion of glycerol to D-xylulose has been achieved using a two stage microbial fermentation. Initially, glycerol was converted to D-arabitol by *Candida parapsilosis*, then yeast cells were collected and finally the D-arabitol from the culture supernatant was converted to D-xylulose by *G. oxydans*[117]. However, this conversion process needs to be improved before increasing production scale since from 170 g/L glycerol only 32.2 g/L of D-arabitol was produced.

### Polyunsaturated fatty acids

Polyunsaturated fatty acids, such has docosahexaenoic acid (DHA, 22:6), eicosapentanoic acid (EPA, 20:5) and α-linoleic acid (ALA, 18:3) have market presence as nutraceuticals, because they are beneficial for human health. The use of biodiesel-derived glycerol as carbon source to produce such polyunsatured fatty acids, especially DHA, was recently reviewed [118,119]. Thus we do not further discuss this route for valorization of crude glycerol in this work.

### Biohydrogen and biomethane

Crude glycerol utilization to produce gaseous fuels, biohydrogen and biomethane (biogas), has gained more attention lately (as reviewed by [119,120]. Indeed, several studies have reported biohydrogen and biomethane production by microbial conversion processes from crude glycerol using single microbial strains or bacterial communities. Utilization of crude glycerol as the sole carbon source or as a suitable co-substrate to improve the efficiency of biodigesters has also been investigated. These topics were currently reviewed and reported in recent publications [119-122] and will not be further discussed here.

### Biodiesel-based biorefinery: opportunities and challenges

Biorefineries are based on the integration of the biomass conversion processes to produce power, fuels and chemicals. In this context, the utilization of glycerol generated in the biodiesel production process offers an excellent opportunity to obtain chemicals by microbial fermentation. Production yields of fuels and chemicals from glycerol as high as 90% of theoretical maximum have been obtained (Table 1). These high production yields of the desired product should make the establishment of bioprocesses easier. Even if the available producing strains have not been evaluated at pilot scale yet (at least based on the publicly available data), market demand for green chemicals, new processes, and technologies for lignocelulose biorefineries should facilitate the development of industrial processes based on crude glycerol.

A vast range of fuels and chemicals can potentially be produced by microbial fermentation of glycerol. However, the expectation is that the primary products obtained by fermentation in biorefineries are chemicals with established demands and international markets. In fact, from the fourteen compounds produced at lab scale by microbial fermentation of glycerol showed in Table 1, ten of them are nowadays produced by chemical or microbial conversion of sugars or petrochemical processes. More interesting, these chemicals are important platform chemicals or products that have a consolidated market demand of thousands of tons per year (Table 2). Thus, once a production process based on glycerol fermentation for any of these chemicals is developed, the product could easily enter the market. On the other hand, it is important that production costs of chemicals from glycerol remain competitive with those obtained from petroleum. It should be noted that demands for sustainable development and volatile petroleum prices should favor the use of “greener” chemicals in the long term, even if their market price is not considerably cheaper. Chemicals without or with a limited market, like DHA and glyceric acid, may represent an opportunity for the development of new products as there is no direct benchmark production price they will need to compete against.

Manipulation of microbes to allow the cost competitive commercial production of fuels and chemicals, such as ethanol, butanol, isoprenoids and others, on sugar and lignocellulose has advanced significantly in the last years [123]. Indeed, several processes based on engineered microorganisms, especially yeast and bacteria species, have been developed and implemented [123]. Two examples of success are given by the production of 1,3-PDO and succinic acid. The chemical 1,3-PDO is traditionally made from fossil-derived ethylene oxide or propylene, however, a bio-based process has been developed and implemented by DuPont and Tate & Lyle. Their process for production of 1,3-PDO relies on a microbe expressing genes from several different microorganisms to give the required productivity [124]. The process based on the use of a designer microbe to produce 1,3-PDO from corn has been running in a Bio-PDO™ plant in Tennessee (USA) with a capacity of 45 thousand tons a year since 2007. Similar approaches are being developed for succinic acid. Bio-based succinic acid production by a fermentative technology is the focus of a joint venture between DSM and Roquette. Indeed, the first testing volumes of this renewable and versatile chemical produced from corn have already been produced in a demonstration plant in Lestrem (France) that was built in 2009. These developments are expected to facilitate the establishment of glycerol-based fermentation processes, since similar microorganisms and pathways may be used.

Although the challenges of obtaining microbial strains able to operate under industrial process conditions have been overcome, another challenge for the production of fuels and chemicals from biodiesel industry crude glycerol is the supply chain. From the economic point of view, it is important for the industry that the feedstocks for biodiesel production, and consequently glycerol for fuels and chemicals production, are abundant year round. In this sense, some countries may not have sufficient biomass, and consequently glycerol, to maintain an industrial-scale production of biodiesel, fuels and chemicals throughout the four seasons, especially due to competition of biomass for other uses. One way to solve this problem, especially in countries with vast territory and mild climate year round, is the diversification of biomass feedstocks in the industry and regionalization of production plants. In this sense, the main feedstock for the industry should be growing in the region of the production plant, which may only be complemented by alternative biomass from other regions. In countries like Brazil, for example, where 80% of biodiesel is produced from soybean oil, it might be advantageous to start using oil from alternative biomass sources, like palm oil, physic nut, castor bean and others. This would guarantee the supply of biomass for the industry and at the same time avoid transportation of soybean to production plants around the country.

## Conclusions

Increasing awareness of environmental issues and consequent pressure from governments and public agencies to reduce the emission of pollutants, together with the increasing petroleum cost and demand for fuels and chemicals worldwide have led to the development of biomass conversion processes. Processes for production of fuels and chemicals from crude glycerol waste from the biodiesel industry have been evaluated and developed at laboratory scale. Indeed, the strong potential of crude glycerol use for the development of biorefineries has been demonstrated by the production of several chemicals using different routes and microorganisms. Several yeasts and bacteria, especially *E. coli* and others from the Enterobacteriaceae and Clostridiaceae families, have been evaluated for fermentation of glycerol. Efficient selection and construction of recombinant strains based on biochemical and genomic data associated with optimization of fermentation conditions resulted in strains able to produce more than 100 g/L of the desired products with yields above 50% of the theorethycal maximum in laboratory scale.

Among the different routes to establish a fermentative process based on microbial fermentation of glycerol, the use of “wild type” natural producers and engineered strains are the most considered. The later case is more common for bacteria of the Enterobacteriaceae family and the former one for engineered strains of yeast and *E. coli*. When wild type strains are employed to produce the desired chemical, efforts to improve production generally concentrate on the optimization of fermentation conditions, like aeration, pH, substrate concentration and feeding. However, the use of these strains (species) in industrial applications may be impaired by pathogenicity, need for strict anaerobic conditions, or lack of genetic tools. The use of engineered strains commonly employed in industry, especially yeasts and *E. coli*, can solve this problem. However, metabolic engineering strategies have to be used to drive metabolite synthesis through homologous and heterologous pathways. Although in industry the most commonly used strains are engineered producers of fuels and chemicals, a careful screening of the biodiversity is generally advisable, since microorganisms can naturally produce a vast range of compounds. Once enzymes and metabolic pathways in such microorganisms are identified and isolated they can be used for the development of recombinant strains.

Considering the already remarkable advances for the production of fuels and chemicals from glycerol using microorganisms, it may be expected that strains for industrial implementation of these bioprocesses should become available in the next years. The technology for the production of butanol, propanodiol and succinic acid from simple renewable feedstocks such as starch and sucrose is already available. Therefore, it is expected that these chemicals will be the first to be produced from more complex substrates such as biodiesel-derived crude glycerol and other more complex biomasses. However, before implementation of a pilot or industrial plant to produce any fuel or chemical, two important aspects should be considered: i) the cost of producing fuels and chemicals by microbial fermentation should be comparable with fossil-derived products; and ii) the feedstock supply chain for the industry.

## Competing interests

JRMA has research grants from the Brazilian National Council for Scientific and Technological Development (CNPq) for experimental investigations on production of fuels and chemicals from glycerol waste of the biodiesel industry.

## Authors’ contributions

LCLF wrote the parts concerning polyols. BFQ participated in the design of the review and revised the manuscript. JRMA participated in the design of the review and wrote most of the manuscript. All authors read and approved the final version.
